# Among common neuropsychological tests, the Paced auditory serial addition test is the strongest predictor of trait fatigue in patients with traumatic brain injury

**DOI:** 10.1111/jnp.12419

**Published:** 2025-02-13

**Authors:** Nils Berginström, Johan Thelander, Peter Nordström, Anna Nordström

**Affiliations:** ^1^ Department of Community Medicine and Rehabilitation Umeå University Umeå Sweden; ^2^ Department of Psychology Umeå University Umeå Sweden; ^3^ Sundsvall‐Härnösand County Hospital Sundsvall Sweden; ^4^ Department of Public Health and Caring Sciences, Clinical Geriatrics Uppsala University Uppsala Sweden; ^5^ School of Sport Sciences UiT the Arctic University of Norway Tromsø Norway; ^6^ Department of Medical Sciences, Health Sciences Uppsala University Uppsala Sweden

**Keywords:** attention, executive function, mental fatigue, neuropsychology, traumatic brain injury

## Abstract

Fatigue is one of the most common symptoms following traumatic brain injury (TBI). Despite its prevalence, fatigue remains a challenging concept to define and measure. The aim of the present study was to explore potential relationships between self‐rated fatigue in patients with TBI and performance on several widely used neuropsychological tests. In a cross‐sectional design, patients with TBI (*n* = 68) and healthy controls (*n* = 27) underwent a comprehensive battery of commonly used neuropsychological tests and completed two self‐assessment fatigue scales, the Fatigue Severity Scale and the Mental Fatigue Scale. Patients with TBI performed worse on neuropsychological tests of short‐term memory, processing speed and executive functioning (inhibition) compared to healthy controls. Within the TBI group, only the Paced Auditory Serial Addition Test (PASAT) and the Stroop—Inhibition task showed significant correlations with measures of fatigue. However, after adjusting for relevant demographic variables, including age, gender, education and TBI severity, only PASAT remained significantly associated with the Mental Fatigue Scale (*r* = .45, *p* = .005). Within the healthy control group, no such associations were found. These results highlight an interesting relationship between PASAT performance and self‐assessed fatigue. With further research, PASAT, modifications of it or similar measures could potentially help clinicians in evaluating fatigue in patients with TBI.

## INTRODUCTION

In patients with traumatic brain injury (TBI), the prevalence of fatigue has been reported to be as high as up to 75% (Andelic et al., 2021; Bushnik et al., 2008; Cantor et al., 2008; Ouellet & Morin, 2006). Fatigue is a multidimensional concept that is difficult to define. It may be of both cognitive and physical nature and is therefore distinguished as central or peripheral (Chaudhuri & Behan, 2000; Leavitt & DeLuca, 2010). Fatigue can also be divided in state or trait fatigue (Genova et al., 2013). State fatigue refers to a condition fluctuating over time due to situational factors, while trait fatigue refers to a relatively stable state that is not likely to change significantly over time. Thus, trait fatigue affects people in everyday life to a larger degree.

Trait fatigue is mostly measured with different self‐rating scales, and objective tests have rarely been used (Johansson, 2021). However, objective measurements of such a disabling symptom as fatigue might be essential in insurance claims or when patients seek support from health care systems, such as sick leave or disability benefits. Studies trying to capture cognitive function and linking this to self‐ratings of trait fatigue have largely been inconclusive. Subjective fatigue have in some studies been associated with poor performance on neuropsychological test in patients with TBI, but the association to objectively measured neuropsychological performance during mental exhausting tasks has been contradictory in different studies and is still unclear (Ashman et al., 2008; Johansson, 2021; Ziino & Ponsford, 2005, 2006b).

This partly inconclusive state of knowledge and the lack of a gold standard for objective clinical measures of fatigue have for a long time been quite an inconvenient fact for both patients and professionals, especially since fatigue is the most commonly reported symptom among patients with TBI (Bushnik et al., 2008; Cantor et al., 2008; Ouellet & Morin, 2006). Researchers have turned toward adjustments of established neuropsychological tests (Möller et al., 2014), new computer‐based tests (Johansson & Rönnbäck, 2015), measuring eye‐movements (oculomotor saccades) (Ettenhofer et al., 2018; Ettenhofer & Barry, 2016; Möller et al., 2019) or new brain imaging techniques to capture fatigue (Berginström et al., 2018). While these recent approaches await replication and further exploration, an objective measure commonly used in clinical practice that can link patients' experience of fatigue is still missing.

The present study aimed to investigate whether there are relationships between self‐reported trait fatigue in TBI patients and the results of widely used neuropsychological tests. Specifically, we sought to determine whether any of these commonly used tests can predict, and serve as an objective measure of, self‐reported fatigue following TBI.

## MATERIALS AND METHODS

### Participants

Ethical approval was obtained from the Regional Ethics Review Board in Umeå, Sweden, and the study was carried out in compliance with the Declaration of Helsinki. Patients with TBI were recruited from a previous clinical trial (Berginström et al., 2017) set at the Neurorehabilitation Clinic at Umeå University Hospital, Umeå, Sweden. A description of the recruitment process for patients, including power analysis, can be found in detail in Berginström et al. (2017). Briefly, inclusion criteria were TBI >12 months previously; age 18–65 years; moderate disability or better recovery on the Extended Glasgow Outcome Scale (Teasdale et al., 1998); and fatigue, defined as a Fatigue Severity Scale (Krupp et al., 1989) score >36. Exclusion criteria were other neurologic or psychiatric conditions, substance abuse, heart‐, liver‐, kidney‐ or active neoplastic disease, and a history of seizures. A total of 68 TBI patients were included according to these criteria.

Healthy controls were recruited via contact with friends and family of patients included in the study, thus a convenience sample. The exclusion criteria for controls were identical to those for patients with TBI, with the addition of never having experienced a concussion or more severe TBI and not suffering from fatigue. From the 59 individuals who reported interest to participate, 30 were selected to match the patient group as closely as possible in terms of gender, age and education. Four of these declined participation before enrolment in the study and were replaced by four other controls.

Sixty‐eight patients with TBI and 30 controls underwent all examinations in the study. During structural magnetic resonance image acquisition for the study by Berginström et al. (2018), one man in the control group showed incidental findings and was thus not included in the present study. Two women in the control group were excluded due to scores above cut‐offs for clinical conditions regarding both fatigue (MFS) and/or anxiety/depression (HADS). Thus, a total of 68 patients with TBI and 27 controls were included in the analyses of the present study.

### Procedure

Before entering the study, all participants read and signed informed consent, during an initial medical examination. Both patients and controls underwent a neuropsychological assessment by a psychologist trained for the task. The tests were administered in the order stated below under Instruments. Self‐assessment scales were completed after participants had finished the neuropsychological assessment. The total testing time for the neuropsychological battery was about 1 h.

### Instruments

#### Self‐assessment scales

*Fatigue Severity Scale* (*FSS*) (Krupp et al., 1989). The FSS is used to assess both the behavioural consequences and the impact of fatigue on daily functioning. The scale consists of nine items, such as ‘Fatigue causes frequent problems for me’, ‘Fatigue interferes with my physical functioning’ and ‘Fatigue interferes with carrying out certain duties and responsibilities’. The FSS have shown acceptable internal consistency (Valko et al., 2008) and test–retest reliability (Mattsson et al., 2008), and studies of validity have shown that it distinguishes well between patients and non‐injured control participants (Krupp et al., 1989; LaChapelle & Finlayson, 1998). FSS have been suggested to be correlated with attention measures in patients with multiple sclerosis (Weinges‐Evers et al., 2010) and executive functioning in patients with stroke (Carrick et al., 2024).

*Mental Fatigue Scale* (*MFS*) (Johansson et al., 2010) is a scale developed in Swedish and is based on the definition of mental fatigue suggested by Johansson (2014). The MFS consists of 15 questions with a cut‐off score of 10.5. MFS items include questions regarding mental fatigue, long recovery time, and associated difficulties such as concentration, motivation and memory problems. The scale shows a high degree of internal consistency (Johansson et al., 2010) and discriminant validity (Johansson & Ronnback, 2014) in patients with neurological conditions. MFS have been suggested to correlate well with cognitive measures of information processing speed in patients with different neurological conditions (Johansson & Ronnback, 2014; Jonasson et al., 2018).

*Hospital Anxiety and Depression Scale* (*HADS*) (Zigmond & Snaith, 1983). The HADS is a 14‐item scale containing two separately scored subscales of anxiety and depression, validated in several patient groups (Caci et al., 2003; Härter et al., 2001).

*Rivermead Post Concussion Symptoms Questionnaire* (King et al., 1995) is a short measure consisting of 16 items about common symptoms after concussion and two optional items where the subject can include their own symptoms.

*Rivermead Head Injury Follow‐Up Questionnaire* (Crawford et al., 1996) consists of 10 items about difficulties with everyday activities, relationships and work. It is a validated measure of outcome across the entire range of severity, but particularly after mild to moderate head injury.

#### Neuropsychological measures

Participants underwent an extensive neuropsychological test battery with neuropsychological tests commonly used in clinical practice. The tests were administered in the following order:

*Rey Auditory Verbal Learning Test* (*RAVLT*) (Rey, 1958) is designed as a list‐learning paradigm and evaluates a wide diversity of functions: short‐term auditory‐verbal memory, learning and retention of verbal information. A list of 15 unrelated words is repeated over five different trials and participants are asked to repeat them after each trial. After an additional list of 15 unrelated words is given, the participant is asked to recall the original list of 15 words (short‐term recall) and then once more after 30 min (long‐term recall). The score is the number of correctly remembered words.

*Coding* from the Wechsler Adult Intelligence Scale—Fourth Edition (WAIS‐IV) (Wechsler, 2008) is the Wechsler version of The Digit‐Symbol Substitution Test. The test primarily measures psychomotor processing speed. Participants is presented with a digit‐symbol code key. Below this, rows containing small blank frames under randomly assigned digits 1–9 is presented. During 2 min, the participants is supposed to draw the correct symbol below each number as quickly as possible. The score is the number of squares correctly completed.

*Digit Span* from the Wechsler Memory Scale—Third Edition (WMS‐III) (Wechsler, 1997) is a measure of working memory capacity and simple attention. Digit Span Forward requires repetition of a series of digits of increasing length in the same order as presented. Digit Span Backward requires participant to repeat the series of digits in the reverse order.

*Trail‐Making Test* (*TMT*) from the Delis‐Kaplan Executive Functions System (D‐KEFS) (Delis et al., 2001) consists of a visual cancellation task and a three additional connect‐the‐circle tasks. The basic conditions of this test measure visual scanning, number sequencing and letter sequencing. In the fourth condition, the subject is required to switch between numbers and letters, and this condition measures flexibility or cognitive shifting. The score in each condition is time to completion.

*Paced Auditory Serial Addition Test* (*PASAT*) (Gronwall, 1977) is a neuropsychological test sensitive to cerebral dysfunction, capturing attention, working memory and executive functions. Subjects hear a recorded number string, read aloud by a male voice, and the task is to add the two most recently heard numbers and tell the answer to the test administrator. The test consists of 61 digits (numbers 1–9 presented in a pseudo‐random order). Originally, four trials with an inter‐stimulus‐interval of 2.4, 2.0, 1.6 and 1.2 s, are presented. In the present study, only the 2.4‐s trial was used. The score is the number of correct answers.

*Symbol Search* from the WAIS‐IV (Wechsler, 2008) is a test of mental speed processing and visual search. Over a 2‐min period, participants are tasked with identifying two specific symbols as quickly as possible within a set of five as quickly as possible. The total score is the number of correctly marked symbols within 2 min.

In *Block Span* from the WMS‐III (Wechsler, 1997), the examiner uses a board with ten randomly distributed cubes. The participant is supposed to repeat sequences of increasing length tapped by the examiner. This is performed in two conditions: first in the same (forward) and secondly in reverse (backward) order. The score is the number of completed sequences. This is a test of visual short‐term and working memory.

*The Colour–Word Interference Test* (*CWIT*) from the D‐KEFS (Delis et al., 2001) is based on the Stroop paradigm, which measure the executive function inhibition (Bugg & Crump, 2012; Stroop, 1935). The test consists of coloured words in contradictory colours, where the participant is supposed to name the colours of the words, not to read them. In this study, the Swedish version of the CWIT in D‐KEFS (Delis et al., 2001) was used. The score is the time to completion.

*Verbal Fluency* from the D‐KEFS (Delis et al., 2001) is an executive functions measure, specifically initiation, monitoring, fluency and cognitive shifting. The participant is asked to generate as many words as possible during 1 min starting with a specific letter (word fluency), by category (semantic fluency) and by alternating between two categories (Category Switching). Scores are the correct number of words in each trial.

### Statistical analyses

All statistical analyses for self‐assessment scales and neuropsychological tests were conducted using SPSS Statistics 23.0.0.3 (IBM, 2015). Differences between patients with TBI and healthy controls were examined using the chi‐squared test for categorical variables. For differences between groups on continuous variables, ANOVAs were used, followed by ANCOVAs when adjusting for relevant covariates. Pearson's correlations were used to investigate if the results of neuropsychological tests and self‐assessed fatigue were correlated within the TBI‐group only. For neuropsychological tests with significant associations to fatigue measures, linear regressions were performed to rule out confounding influence of age, education, gender and TBI severity. All statistical tests were two‐tailed, with a significance level of *p* < .05.

## RESULTS

### Participants

The severity of TBI, judged by physician‐generated diagnosis, varied in the TBI group, with 63% having mild, 22% moderate and 15% severe TBI (Table 1). No differences between patients with TBI and healthy controls were found regarding age and gender. Differences were found in employment status (*p* = .011) and education (*p* = .028) with the control group having a higher proportion of full‐time employment and a higher proportion of participants with university education, while the TBI group had a higher proportion of participants with only primary school education.

**TABLE 1:** Demographical characteristics and self‐assessment scales.

	TBI group (*n* = 68)	Controls (*n* = 27)	*p*‐value
Age (years)	41.5 (13.5)	38.2 (12.3)	.268
Years since injury	8.8 (7.4)		
TBI severity			
Mild	43 (63%)		
Moderate	15 (22%)		
Severe	10 (15%)		
Female gender	27 (40%)	13 (48%)	.452
Education			.028*
Primary school	8 (12%)	0 (0%)	
Vocational school	9 (13%)	2 (7%)	
Upper secondary school	29 (43%)	9 (33%)	
Higher education/university	19 (28%)	16 (59%)	
Employment			.011*
Sick leave/retired	13 (27%)	1 (4%)	
Full‐time employee	18 (37%)	20 (74%)	
Part‐time employee	9 (18%)	3 (11%)	
Full‐time student	4 (8%)	3 (11%)	
Part‐time student	2 (4%)	0 (0%)	
Fatigue Severity Scale	47.9 (8.4)	24.1 (4.6)	<.001***
Mental Fatigue Scale	18.3 (5.3)	3.9 (3.5)	<.001***
Hospital Anxiety and Depression Scale—Depression	6.2 (3.4)	2.0 (1.5)	<.001***
Hospital Anxiety and Depression Scale—Anxiety	7.5 (4.0)	4.5 (2.4)	<.001**
Rivermead Post‐Concussion Symptoms Questionnaire	27.6 (10.3)	8.0 (6.3)	<.001***
Rivermead Head Injury Follow‐Up Questionnaire	22.6 (10.0)	2.4 (5.1)	<.001***

*Note*: Data are mean (SD) or number (%).

Abbreviation: TBI, traumatic brain injury.

* *p* < .05.

** *p* < .01.

*** *p* < .001.

### Self‐assessment scales and neuropsychological test results

Patients with TBI scored higher on all self‐assessment scales compared to the healthy control group, all *ps* < .001 (Table 1). In the unadjusted analysis, the control group scored higher on most neuropsychological tests (Table 2). However, there were no significant differences between groups on scores for PASAT, Block Span total and Verbal Fluency—Word fluency. Adjusting for differences in age and education erased the differences between groups on several neuropsychological tests, but patients still performed significantly lower on RAVLT short‐term recall (*p =* .046), Coding (*p <* .001), TMT Visual Search (*p* = .003), TMT Letter Sequencing (*p* = .001), Symbol Search (*p <* .001) and CWIT Inhibition/Switching (*p <* .001).

**TABLE 2:** Neuropsychological tests.

	TBI group (*n* = 68)	Controls (*n* = 27)	*p*‐value
Rey Auditory‐Verbal Learning Test: learning	44.9 (11.1)	51.7 (8.3)	.002**
Rey Auditory‐Verbal Learning Test: short‐term recall	9.0 (3.5)	11.4 (2.6)	.001**
Rey Auditory‐Verbal Learning Test: delayed recall	9.0 (3.5)	11.2 (2.7)	.005*
Coding	52.9 (17.2)	75.9 (13.1)	<.001***
Digit Span total	14.8 (3.4)	16.6 (3.4)	.030*
Trail‐Making Test: Visual Search^a^	21.6 (6.6)	16.3 (4.4)	<.001**
Trail‐Making Test: Number Sequencing^a^	32.9 (9.2)	23.9 (9.2)	.012**
Trail‐Making Test: Letter Sequencing^a^	31.6 (14.6)	21.6 (7.9)	.001**
Trail‐Making Test: Number‐Letter Switching^a^	105.9 (77.6)	62.9 (18.1)	<.001**
Paced Auditory Serial Addition Test^b^	44.2 (10.8)	47.6 (9.2)	.175
Symbol Search	28.5 (8.7)	40.4 (7.0)	<.001***
Block Span total	17.2 (3.1)	17.9 (3.0)	.324
Colour–Word Interference Test: Inhibition	62.2 (24.8)	50.4 (12.8)	.003**
Colour–Word Interference Test: Inhibition/switching	81.6 (34.0)	53.3 (14.5)	<.001***
Verbal Fluency: Word Fluency	40.4 (13.6)	44.8 (14.1)	.168
Verbal Fluency: Category Fluency	45.3 (12.4)	50.9 (10.5)	.041*
Verbal Fluency: Category Switching	13.7 (2.9)	15.3 (2.7)	.016*

*Note*: Data are mean (SD).

Abbreviation: TBI, traumatic brain injury.

^a^ *n*_TBI_ = 67.

^b^ *n*_TBI_ = 50.

* *p* < .05.

** *p* < .01.

*** *p* < .001.

**FIGURE 1:**
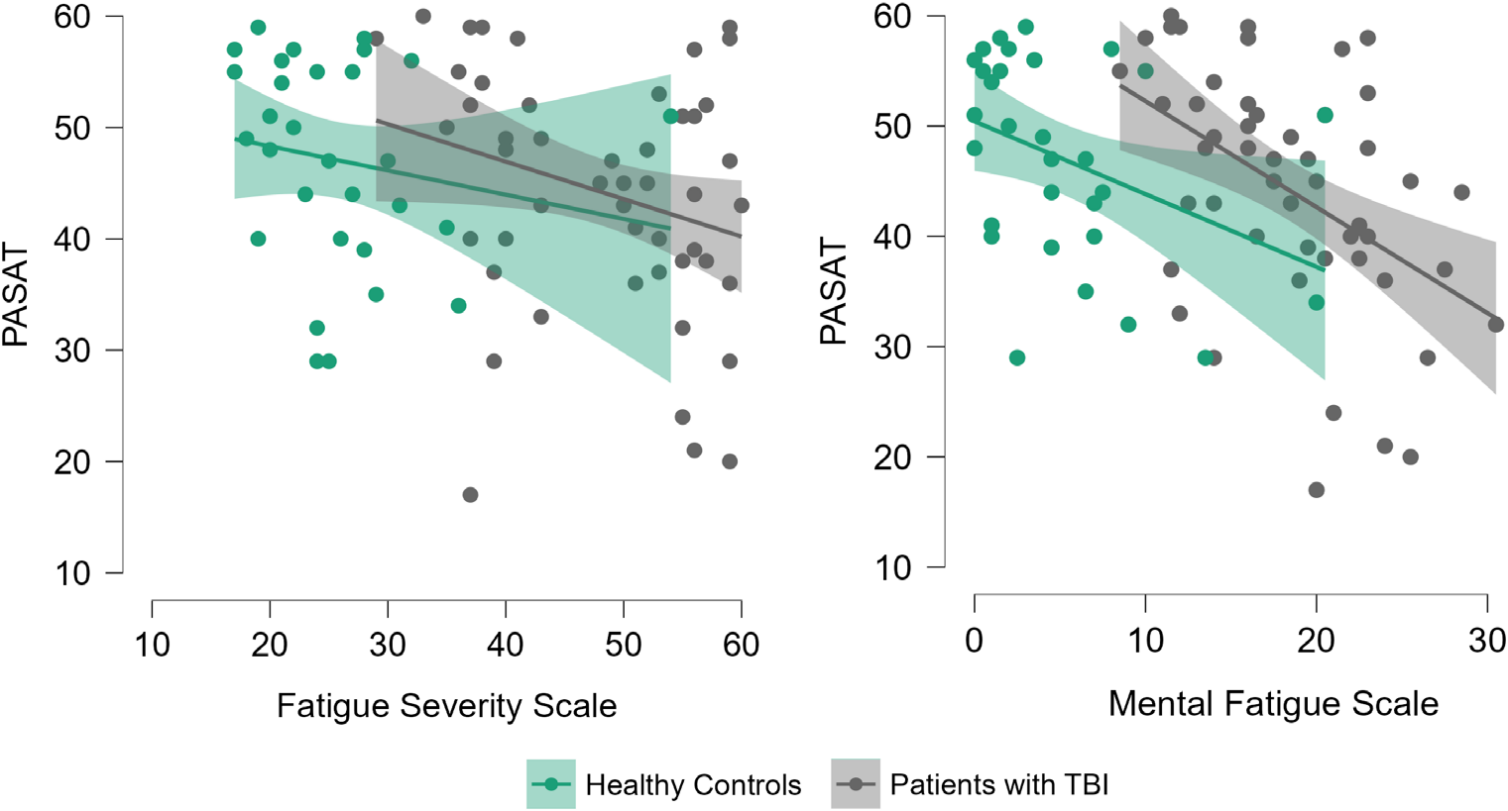
Results from PASAT and the self‐assessment scales of fatigue in patients with TBI and healthy controls.

Within the TBI patient group, only PASAT and Stroop—inhibition correlated significantly with self‐assessed fatigue within the TBI group (Table 3). Stroop—inhibition showed a small positive correlation with FSS, (*r* = .27, *p* = .027) and MFS (*r* = .30, *p* = .012). PASAT had a moderately negative correlation with MFS, (*r* = −.47, *p* = .002), but no significant correlation to FSS (Figure 1). PASAT was also significantly correlated to MFS within the healthy control group in the unadjusted analysis (*r* = −.39, *p* = .036). There were no other significant correlations between any fatigue measure and neuropsychological test within the healthy control group. In all cases, significant correlations indicated that worse performance was associated with a higher degree of fatigue, since the Stroop score is based on time to complete the task and PASAT is the total score of correct answers within the time limit.

**TABLE 3:** Correlations between Mental Fatigue Scale, Fatigue Severity Scale and neuropsychological tests in the TBI patient group.

	FSS	MFS
Rey Auditory‐Verbal Learning Test: learning	.07	−.05
Rey Auditory‐Verbal Learning Test: short‐term recall	.04	−.04
Rey Auditory‐Verbal Learning Test: delayed recall	.08	.04
Coding	−.08	−.11
Digit Span total	−.10	−.11
Trail‐Making Test: Visual Search^a^	−.01	.10
Trail‐Making Test: Number Sequencing^a^	.18	.16
Trail‐Making Test: Letter Sequencing^a^	.12	.14
Trail‐Making Test: Number‐Letter Switching^a^	.09	.22
Paced Auditory Serial Addition Test^b^	−.27	−.47***
Symbol Search	−.01	−.10
Block Span total	−.20	−.11
Colour–Word Interference Test: Inhibition	.27*	.30*
Colour–Word Interference Test: Inhibition/switching	.12	.13
Verbal Fluency: Word Fluency	−.09	−.12
Verbal Fluency: Category Fluency	−.11	−.06
Verbal Fluency: Category Switching	−.09	−.00

*Note*: Pearson's correlation, two‐tailed, *n* = 68.

Abbreviations: FSS, Fatigue Severity Scale; MFS, Mental Fatigue Scale.

^a^ *n* = 67.

^b^ *n* = 50.

* *p* < .05.

*** *p* < .001.

Linear regressions were conducted to adjust for the influence of age, education, gender and TBI severity on the significant correlations. Subsequently, Stroop—inhibition was no longer significantly associated with either FSS or MFS. However, the association between PASAT and MFS remained significant in the fully adjusted model (*r* = −.45, *p* = .005). Subgroup analyses within the TBI patient group, revealed that this association only were present within the mild TBI group (*r* = −.56, *p* = .003) after adjusting for age, education and gender, while no significant associations were found within the moderate or severe TBI groups. The association between PASAT and MFS within the healthy control group was also no longer significant after adjusting for age, education and gender (*p* = .279) (Table 4).

**TABLE 4:** Full regression model with Mental Fatigue Scale as outcome and PASAT as predictor in the TBI patient group.

	*B*	Std. error	Beta	*t*	*p*
(Constant)	31.04	5.30		5.86	<.001
PASAT	−.22	.07	−.45	−2.98	.005
Age	−.02	.06	−.05	−.35	.725
Education	−.19	.94	−.03	−.20	.845
Gender	−2.78	1.51	−.27	−1.84	.074
TBI severity	−.09	1.07	−.01	−.09	.931

## DISCUSSION

The purpose of the present study was to investigate whether there are relationships between self‐reported trait fatigue in TBI patients and the results of widely used neuropsychological tests. In line with previous research (Azouvi et al., 2017; Dikmen et al., 1995; Slovarp et al., 2012; van der Naalt et al., 1999), patients with TBI performed worse than healthy controls on neuropsychological tests of short‐term memory, processing speed and executive functioning (Kennedy et al., 2009), although several differences disappeared after adjusting for age and education. In a broad neuropsychological battery measuring working memory, attention, processing speed, executive functions and various aspects of memory, only the PASAT and Stroop inhibition were found to significantly correlate with fatigue within the patient group. After adjustment for relevant demographic and injury‐related factors, only PASAT remained significantly associated with fatigue. This suggests that the cognitive functions assessed by PASAT may be linked to trait fatigue in TBI patients, and future studies on this relationship is warranted.

The observed association between PASAT performance and self‐rated fatigue aligns with its original purpose of identifying cognitive impairments post‐TBI (Gronwall, 1977). Tombaugh (2006) summarizes that PASAT is a highly sensitive but non‐specific measure of cognitive impairments, not restricted to measuring cognitive processes related to attention. Despite concerns regarding PASAT's specificity in assessing TBI severity (Sherman et al., 1997), our findings underscore its sensitivity to cognitive processes such as attention, concentration and information processing capacity. The widespread activation of brain regions during PASAT performance, as evidenced by neuroimaging studies (Lockwood et al., 2004), may explain its predictive utility for mental fatigue post‐TBI. This may also be interpreted as being in line with Chaudhuri and Behan's (2000) theory of the role of the basal ganglia and dysfunction in striato‐thalamic‐cortical loops in central fatigue. One of the mechanisms of striato‐thalamic‐cortical loops is to regulate vast spread activation of the brain, and dysfunction in these loops is closely related to fatigue in TBI (Berginström et al., 2018) as well as other neurological diseases such as multiple sclerosis (Engstrom et al., 2013; Filippi et al., 2002), Parkinson's disease (Pavese et al., 2010), chronic fatigue syndrome (Miller et al., 2014) or stroke (Dobryakova et al., 2013; Tang et al., 2013; Wei et al., 2016).

The mechanisms underlying persistent mental fatigue in TBI patients are multifaceted and may involve reduced cellular energy due to inflammation (Lacourt et al., 2018), as well as increased cognitive effort to compensate for impairments in attention, processing speed and working memory (van Zomeren & van den Burg, 1985). The latter, named the coping hypothesis, has been supported in several studies (Belmont et al., 2006, 2009; Ziino & Ponsford, 2006a, 2006b). These two mechanisms do not conflict in their slightly different conceptual levels and can be present independently or simultaneously. Considering the high cognitive load that PASAT imposes on subjects, the results are in line with the coping hypothesis. This theory suggests that the brain works harder compensating for deficits in cognitive functions such as processing speed, attention and working memory, which results in fatigue (van Zomeren & van den Burg, 1985). In the case of the results from this study, this is not only about fatigue in the moment (state fatigue), but also about fatigue in everyday life (trait fatigue), possibly due to constant compensation for cognitive impairments.

The combination of the PASAT being able to predict self‐assessed trait fatigue and at the same time not being able to distinguish TBI patients from controls is a fact in this study that is difficult to understand from a clinical perspective. This might be an effect of the way scores are counted in the PASAT, simply in sequence, and the subjects may skip numbers in pairs as a way to manage and therefore get a higher score. Still, PASAT was only robustly associated to fatigue within the TBI patient group. This indicates that it is only in persons suffering from fatigue, that the cognitive processes required to complete the PASAT is associated with fatigue. Furthermore, our findings indicate that while TBI patients exhibited poorer performance on most neuropsychological tests assessing short‐term memory, processing speed and executive functioning compared to healthy controls, many of these differences diminished after adjusting for age and education. This underscores the importance of considering these demographic factors in cognitive assessments, and when examining correlates of these assessments.

Another interesting aspect of the results from the present study is that PASAT was only associated with MFS and not to the FSS. This is likely due to the fact that these two scales measure different forms of fatigue. FSS focus on physical fatigue and consequences of fatigue in everyday life (Krupp et al., 1989), while the MFS was specifically developed to capture mental fatigue in neurological conditions (Johansson et al., 2010). Fatigue is a multidimensional concept that can be defined in various ways, such as mental or physical fatigue (Leavitt & DeLuca, 2010), central or peripheral fatigue (Chaudhuri & Behan, 2000, 2004) and state or trait fatigue (Genova et al., 2013). The results of the present study pertain to trait mental fatigue. Future studies should carefully consider which types of fatigue are being investigated and identify variables that is associated with the specific form of fatigue under study.

### Limitations and future directions

One major limitation of this study is the heterogeneous sample of patients, ranging from mild to severe TBI. Another drawback of this study is the fact that 18 patients with TBI did not manage to perform the PASAT. It is hard to determine how many of the patients were unable to understand the instructions, unable to perform the test, or were unable to perform it due to emotional distress. The test is known to raise anxiety among patients and controls (Tombaugh, 2006). Since the sample is a clinically representative sample of TBI patients, other tests that are similar to the PASAT might be more suitable to evaluate in future studies and in the clinical neuropsychological practice. Alternatives to the PASAT, where the numbers have been exchanged with words, such as the Paced Auditory Serial Opposites Task, PASOT (Gow & Deary, 2004), have been explored and shown to raise less anxiety when performed and still show a high correlation to PASAT scores. To our knowledge, studies using PASOT have not yet been replicated or further explored in clinical populations. Other approaches, such as dividing PASAT/PASOT into intervals, and investigating performance changes over time might be another possible method. Some studies have done this by modifying standard tests of mental speed and attention (Holmqvist et al., 2024; Moller et al., 2014), but not to our knowledge in more complex tests such as PASAT or n‐back. Others have developed similar but completely new computer‐based tests (Johansson & Ronnback, 2015). Still, these approaches await replication and further exploration, and more research within the field of fatigue after TBI but is indeed of high interest.

## CONCLUSIONS

In summary, our study underscores the significance of the Paced Auditory Serial Addition Test (PASAT) as a robust predictor of self‐rated trait fatigue among TBI patients, even after adjusting for various demographic and injury‐related factors. The observed link between PASAT performance and fatigue is consistent with its intended use in identifying cognitive impairments following TBI, highlighting its sensitivity to cognitive processes like attention and information processing. Our findings also lend support to the coping hypothesis, suggesting that increased cognitive effort may contribute to persistent mental fatigue in TBI patients. The PASAT is a brief (2–3 min) neuropsychological test that is easy to administer, score and interpret. The test may, however, as shown by the high level of attrition in this study, still be too challenging for some patients, especially with a higher degree of cognitive difficulties. With further research, PASAT may be useful in evaluating fatigue in both patients with TBI and other conditions, such as multiple sclerosis, stroke or post‐COVID‐19.

## AUTHOR CONTRIBUTIONS

**Nils Berginström:** Conceptualization; methodology; data curation; investigation; formal analysis; supervision; project administration; writing – original draft; writing – review and editing. **Johan Thelander:** Conceptualization; formal analysis; writing – original draft; writing – review and editing. **Peter Nordström:** Conceptualization; methodology; formal analysis; funding acquisition; supervision; writing – review and editing. **Anna Nordström:** Conceptualization; methodology; investigation; supervision; funding acquisition; writing – original draft; writing – review and editing.

## CONFLICT OF INTEREST STATEMENT

The authors have no conflict of interest.

## Data Availability

The data that support the findings of this study are available on request from the corresponding author. The data are not publicly available due to privacy or ethical restrictions.
